# INCREASED HSP25 DRIVES THE TRANSITION FROM PROTEASOME TO AUTOPHAGY-MEDIATED DEGRADATION UNDER PROTEOTOXIC STRESS

**DOI:** 10.1093/geroni/igz038.371

**Published:** 2019-11-08

**Authors:** Jessica L Scheirer, Karl Rodriguez

**Affiliations:** 1 UT Health Science Center, San Antionio, San Antionio, Texas, United States; 2 UT Health San Antonio, San Antonio, Texas, United States

## Abstract

Accumulation of protein aggregates are a common pathology in many neurodegenerative disorders. This accumulation may be due to a function decline in the protein homeostasis network known to occur during the aging process. Small heat shock proteins are a class of molecular chaperones that assist in protein folding and ameliorates the degradation activity of the proteasome and autolysosome thereby decreasing disease-associated aggregates. Prior work in rodents and C. elegans has shown expression levels of the small heat shock protein 25 (HSP25) correlates with maximum lifespan potential. Increased levels of HSP25 extends lifespan in a transgenic C. elegans model. This lifespan extension is dependent on skn-1 with evidence suggesting an enrichment in several skn-1-related pathways, such as lysosomal genes. Concomitantly, proteasome activity declines while autolysosome activity increases. This observation might suggest a switch from proteasome degradation to autophagy as the main driver of protein degradation in C. elegans in this transgenic model. To investigate if a reduction of proteasome function and elevated lysosomal gene activation during aging and under proteotoxic stress are modulated by HSP25 we have crossed our HSP25-transgenic worm with an aggregating and non-aggregating tau worm model. This work will elucidate a possible mechanism that explains the change in the protein degradation response pathways potentially modulated by HSP25 during increased protein misfolding.

